# Identification of CCL4 as an Immune-Related Prognostic Biomarker Associated With Tumor Proliferation and the Tumor Microenvironment in Clear Cell Renal Cell Carcinoma

**DOI:** 10.3389/fonc.2021.694664

**Published:** 2021-11-24

**Authors:** Lu Zhang, Mengzhao Zhang, Lu Wang, Jianlong Li, Tao Yang, Qiuya Shao, Xiao Liang, Minghai Ma, Nan Zhang, Minxuan Jing, Rundong Song, Jinhai Fan

**Affiliations:** ^1^ Department of Urology, The First Affiliated Hospital of Xi’an Jiaotong University, Xi’an, China; ^2^ Department of Urology, Xi’an NO.3 Hospital, The Affiliated Hospital of Northwest University, Xi’an, China; ^3^ Oncology Research Lab, Key Laboratory of Environment and Genes Related to Diseases, Ministry of Education, Xi’an, China

**Keywords:** CCL4, tumor proliferation, immune infiltration, tumor microenvironment, immune checkpoints, clear cell renal cell carcinoma

## Abstract

The last decade has witnessed revolutionary advances taken in immunotherapy for various malignant tumors. However, immune-related molecules and their characteristics in the prediction of clinical outcomes and immunotherapy response in clear cell renal cell carcinoma (ccRCC) remain largely unclear. C-C Motif Chemokine Ligand 4 (CCL4) was extracted from the intersection analysis of common differentially expressed genes (DEGs) of four microarray datasets from the Gene Expression Omnibus database and immune-related gene lists in the ImmPort database using Cytoscape plug-ins and univariate Cox regression analysis. Subsequential analysis revealed that CCL4 was highly expressed in ccRCC patients, and positively correlated with multiple clinicopathological characteristics, such as grade, stage and metastasis, while negatively with overall survival (OS). We performed gene set enrichment analysis (GSEA) and gene set variant analysis (GSVA) with gene sets coexpressed with CCL4, and observed that gene sets positively related to CCL4 were enriched in tumor proliferation and immune-related pathways while metabolic activities in the negatively one. To further explore the correlation between CCL4 and immune-related biological process, the CIBERSORT algorithm, ESTIMATE method, and tumor mutational burden (TMB) score were employed to evaluate the tumor microenvironment (TME) characteristics of each sample and confirmed that high CCL4 expression might give rise to high immune cell infiltration. Moreover, correlation analysis revealed that CCL4 was positively correlated with common immune checkpoint genes, such as programmed cell death protein 1 (PD-1), cytotoxic T-lymphocyte-associated protein 4 (CTLA4), and lymphocyte activating 3 (LAG3). Overall, this study demonstrated that CCL4 might serve as a potential immune-related prognostic biomarker to predict clinical outcomes and immunotherapy response in ccRCC. Moreover, CCL4 might contribute to TME modulation, indicating the mechanism CCL4 involved in tumor proliferation and metastasis, which could provide novel therapeutic perceptions for ccRCC patients.

## Introduction

Kidney cancer ranks among the top 10 cancer killers (1), while malignant kidney tumors contribute to 2% of the cancer burden in the world with increasing incidence (2). In 2020, 73,750 new cases and 14,830 deaths of kidney cancer were predicted to occur in the US, equivalent to approximately 202 new cases and 41 deaths per day (3). Clear cell renal cell carcinoma (ccRCC) is the dominant histological subtype of kidney cancer, accounting for nearly 75% of all cases (4). Although considerable research has focused on exploring mechanisms of ccRCC progression, the specific etiology and carcinogenic process are ill-defined.

In the background of chromosome 3p loss, there is frequent loss of heterozygosity in four aberrant tumor suppressor genes: Von Hippel-Lindau Tumor Suppressor (VHL), Polybromo 1 (PBRM1), SET Domain Containing 2 (SETD2), and BRCA1 Associated Protein 1 (BAP1) within ccRCC. VHL mutation is the most common event among all mutations, which stimulates vascular endothelial growth factor (VEGF) to proceed angiogenesis and tumor proliferation by modulating the stabilization of hypoxia-inducible factor (HIF) 1α and 2α (5). A previous study (6) suggested that the aberrantly altered genes in ccRCC also included PI3K/mTOR pathway genes (MTOR, PTEN, and PIK3C), the NRF2-ARE pathway gene NFE2L2A, the HIPPO pathway gene NF2, etc (7). Therefore, several small molecule agents targeting VEGF or mTOR have been approved for clinical use, such as bevacizumab, sunitinib, cabozantinib, everolimus and temsirolimus (8). However, most of these agents are multitargeted resulting in multiple drug resistance and serious adverse effects. Notably, a recent study identified a mixed subgroup in ccRCC with comprehensive bioinformatics tools. The patients in this mixed group were characterized by upexpression of mitochondrial and weakened angiogenesis-related genes, making them a distinct therapy stratification compared to the traditional ccRCC patients (9). Recently, with the advent of immunotherapy, a new era of immune checkpoint inhibitors (ICIs) has broadened the treatment landscape of kidney cancer, especially ccRCC, which is widely considered to be sensitive to immunotherapy (10–12). To date, several clinical trials have proposed that antiangiogenics combined with immunotheraputic strategies can achieve greater therapeutic efficacy compared with traditional tyrosine kinase inhibitors targeting VEGF or mTOR inhibitors alone, which has become an alternative first-line treatment for ccRCC in the clinic, emphasizing the crucial status of the tumor microenvironment (TME) (13, 14).

The TME is comprised of various components: immune cells, fibroblasts, endothelial cells, hormones, cytokines, the extracellular matrix, etc. surrounded by tumor cells and the vasculature (14, 15). A previous study (16) has shed light on the insights that the TME can not only have profound effects on tumor proliferation and metastasis, but also closely correspond to therapeutic efficacy (17–19). Despite the unprecedented favorable outcomes achieved with wide immunotherapy use in various cancers, the majority of patients treated with immunotherapeutic strategies still do not achieve long-term positive response rates attributing to primary, adaptive or acquired resistance (20). Sharma et al. suggested that the tumor cell-extrinsic mechanism of immunotherapy resistance is closely related to various immune cells within the immunosuppressive TME, which may release factors that inhibit antitumor immunity into the circulation to stimulate immune checkpoints and exhaust cytotoxic T cells (CTLs), including CTLA4 and antibodies against PD-1 (21–23). According to previous studies (24, 25), the mechanism in which regulatory T cells could secrete suppressive immune-related cytokines, while myeloid and stromal cells could stimulate immune inhibitory checkpoints (PD-1, CTLA4 and TIM-3) has been adopted by many cancers to evade immune surveillance (24–26), which contributes to aggressive tumor proliferation and low ICI efficacy. Therefore, it is urgent to distinctly clarify the underlying mechanism of TME modulation in tumor progression and immunotherapy response, which might uncover promising therapeutic targets for ccRCC patients (27, 28).

Taken together, tyrosine kinase inhibitors targeting VEGF or mTOR inhibitors are palliative, while ICIs are restricted to only a few patients for immunotherapy resistance in the long term, attributing to complex biological processes within the immunosuppressive TME. However, to the best of our knowledge, few studies have focused on the molecular characteristics and comprehensive set of enriched pathways of a reliable immune-related prognostic biomarker involved in TME modulation in ccRCC. In this study, we identified a potential immune-related candidate, CCL4, associated with remodeling the TME derived from the intersection of data from the GEO, TCGA and the ImmPort databases using integrated bioinformatics analysis, which is definitely the first work to imply the molecular characteristics and value of CCL4 in tumor progression, immune infiltration and immunotherapy response in ccRCC.

## Materials and Methods

### Data Acquisition and Processing

Four mRNA microarray datasets (GSE6344, GSE781, GSE15641, and GSE77199) and raw transcriptome sequence data of 606 samples were derived from NCBI GEO database (http://www.ncbi.nlm.nih.gov/geo/) and TCGA data portal (https://portal.gdc.cancer.gov/), respectively. Clinical data downloaded from TCGA were filtered to exclude samples with incomplete clinical characteristics and follow-up information. After rigorous screening, 530 TCGA-KIRC samples were included in this study for further survival analysis. Besides, immune-related gene sets were downloaded from the ImmPort database (https://www.immport.org/home).

### Identification of DEGs

The differentially expressed genes (DEGs) between tumor tissues and matched normal tissues were analyzed using GEO2R(https://www.ncbi.nlm.nih.gov/geo/geo2r), an alternative web tool supported by “limma” R package. With the threshold of |log2 Fold Change| >1 and P value <0.05, 342 common DEGs (240 upregulated genes and 132 downregulated genes) were screened out using the online tool Venny 2.1 (https://bioinfogp.cnb.csic.es/tools/venny/index.html) to visualization.

### Functional Enrichment Analysis

To investigate the underlying biological functions of DEGs, we performed Gene Ontology (GO) and Kyoto Encyclopedia of Genes and Genomes (KEGG) enrichment analyses to explore significant functional pathways through the Database for Annotation Visualization and Integrated Discovery website (DAVID, http://david.ncifcrf.gov) (29), which provides a thorough set of functional annotation tools to identify enriched biological functions. The criteria P value <0.05 and false discovery rate <0.05 were recognized to be statistically significant. The “GOplot” and “ggplot2” package were utilized to visualize GO terms and KEGG pathways. Furthermore, gene set enrichment analysis (GSEA) and gene set variant analysis (GSVA) were conducted by “clusterProfiler” package and “GSVA” package respectively to clarify enriched pathways that significantly altered between high and low CCL4 expression groups (30).

### Survival Analysis

Overall survival analysis of selected genes and VHL mutant/wild-type groups stratified by CCL4 expression were conducted using “survival” and “survminer” package. In addition, univariate Cox regression analysis and Receiver operating characteristic (ROC) curve analysis were employed to extract the potential immune-related prognostic biomarker based on survival analysis. Hazard Ratio >1 was considered to proceed tumor progression while genes with Harzrd Ratio <1 were defined as protectors of ccRCC.

### Evaluation of Immune Infiltration

A deconvolution algorithm developed by Newman et al., “CIBERSORT” algorithm was applied to quantify the relative proportion of 22 immune cell types in different CCL4 expression groups running with provided LM22 signature matrix at 1000 permutations. The outputs were considered to be accurate after screening to meet P value <0.05. In addition, Estimation of Stromal and Immune cells in malignant tumors using Expression data (ESTIMATE) method (27) conducted by the “estimate” package was used to evaluate the infiltration levels of immune cells and stromal cells in the TME of two groups stratified by CCL4 expression, which contains estimate score, immune score, stromal score and tumor purity (27).

### Collection of Somatic Alteration Data

Somatic mutation gene profile, in the form of Mutation Annotation Files (MAF), were imported into R to display somatic mutation landscape of TCGA-KIRC samples. The top 20 most frequently altered genes were extracted using the R package “maftools” (31). To determine tumor mutational burdens of KIRC samples, we calculated non-synonymous mutations based on the stratification of CCL4 expression and conducted TMB score by “TCGAmutations” package.

### Statistical Analysis

All statistical analyses and visualization of results were performed using R software version 4.0.3. The Kruskal-Wallis test was used to compare multiple groups, while the Wilcoxon test was used to compare two groups. The Kaplan-Meier plot was performed to display survival curves for subgroups in various stratified analysis, and the log rank test were used to evaluate statistically differences. Pearson analysis computed correlation coefficient among variances of this study. P value <0.05 was considered to be statistically significant.

## Results

### Identification and Enrichment Analysis of DEGs

**Figure 1:**
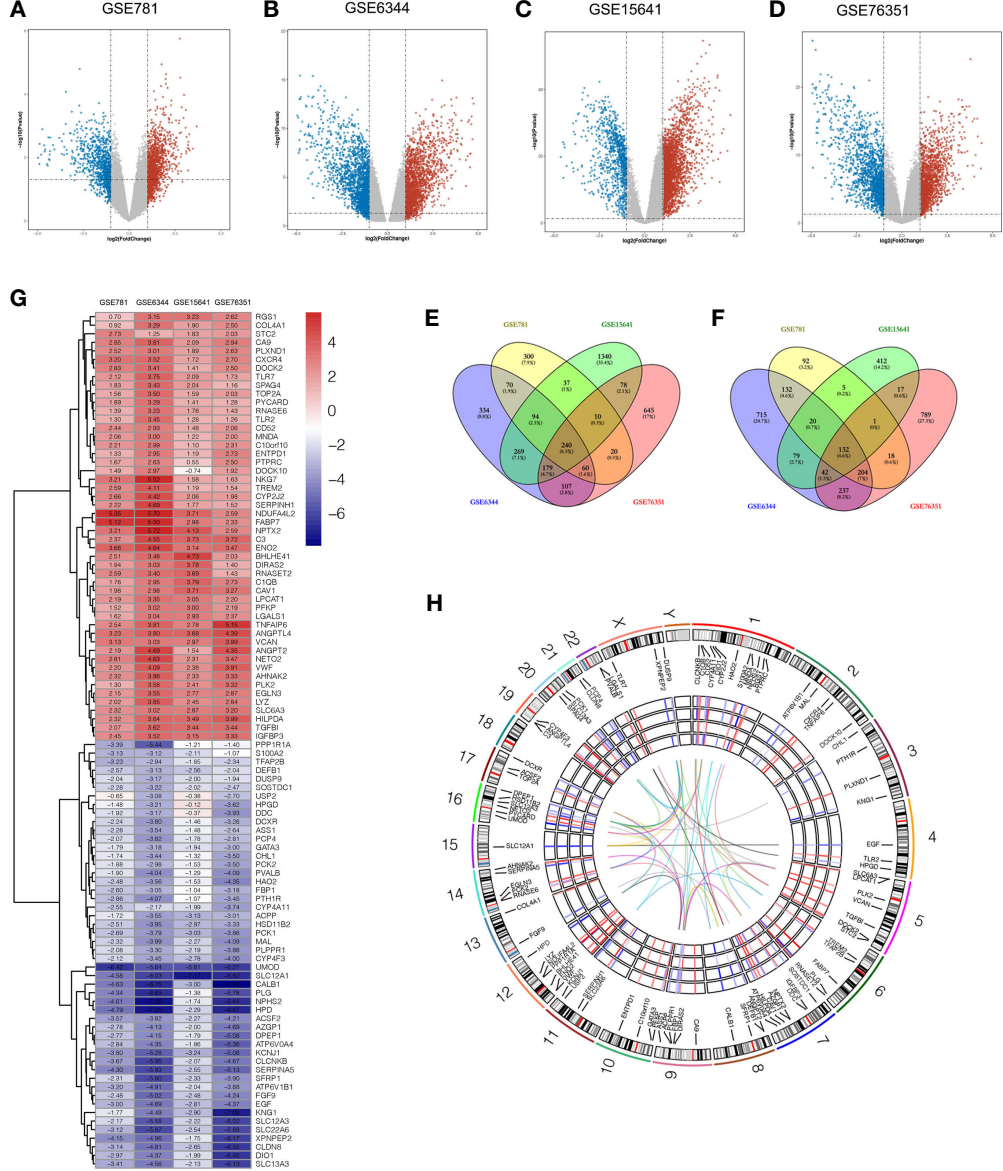
Identification of DEGs between tumor and adjacent normal tissues in four ccRCC microarray datasets. **(A–D)** Volcano plots of GSE781 **(A)**, GSE6344 **(B)**, GSE15641 **(C)**, and GSE76351 **(D)**, from the GEO database. **(E, F)** Venn diagrams of the overlapping DEGs identified in the four datasets. The overlapping areas represent 240 upregulated genes **(E)**, and 132 downregulated genes **(F)**. The cutoff criteria were P value < 0.05 and |log2FC| > 1. **(G)** Heatmap of the top 50 upregulated genes and top 50 downregulated genes of DEGs. Each column represents a dataset and each row represents a gene. The number upon each rectangle is the log2FC value. **(H)** Circular visualization of expression profiles, chromosomal positions and correlation of the top 100 DEGs. The rainbow network in the center corresponds to a strong correlation (Pearson correlation coefficient > 0.8) among the top 100 DEGs. The four GEO datasets expression profiles are presented in the middle circular heatmaps. The gradient ranging from blue to red represents the changing spectrum from down- to upregulation. The outer circle corresponds to 24 chromosomes, and lines deriving from each gene point to their specific chromosomal locations. DEG, differentially expressed genes; ccRCC, clear cell renal cell carcinoma; GEO, Gene Expression Omnibus; log2FC, log2 Fold Change.

**Figure 2:**
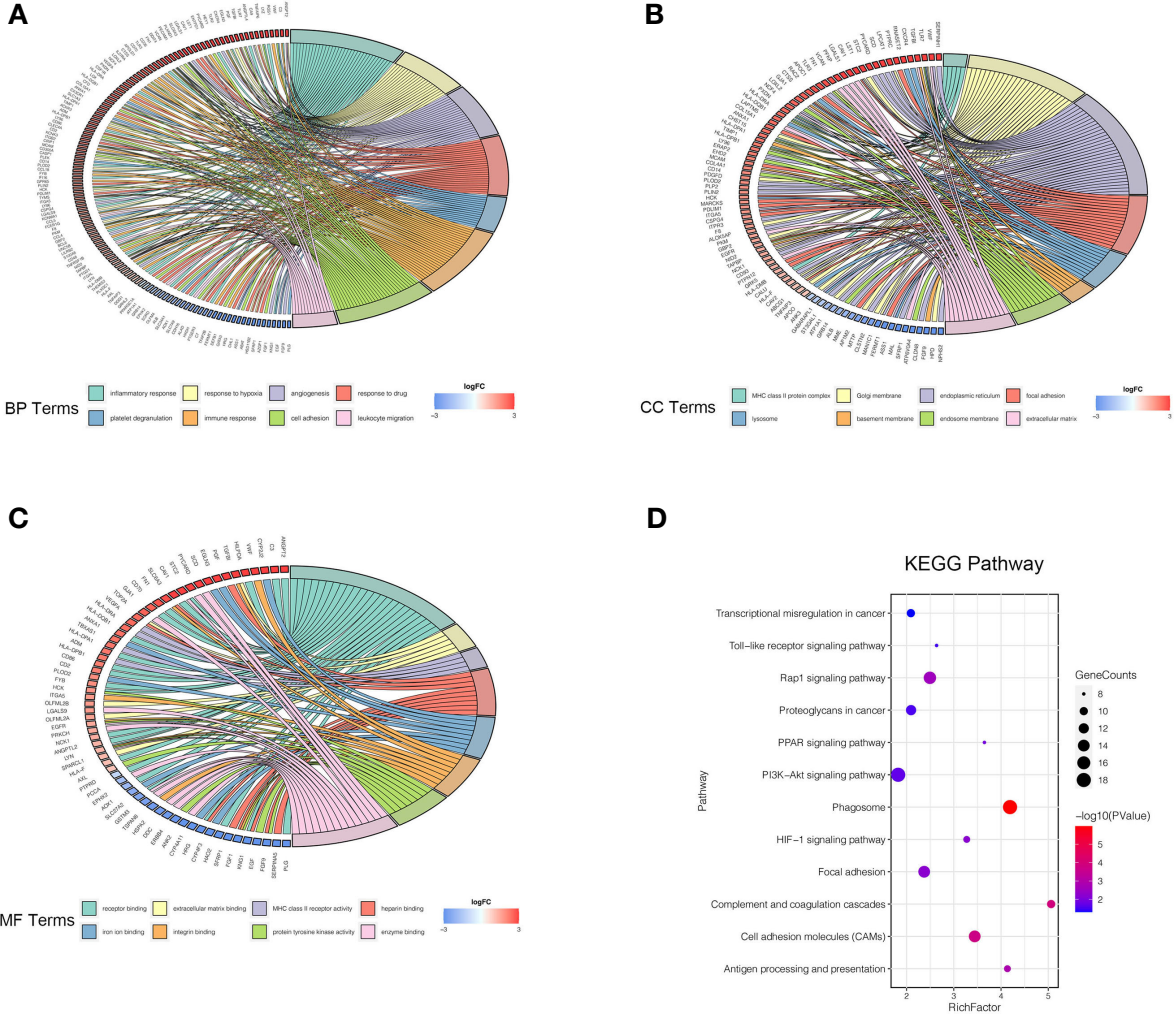
GO annotation and KEGG pathway enrichment analysis of DEGs. **(A–C)** Chord diagrams represent the correlations between DEGs and significantly enriched GO terms: BP **(A)**, CC **(B)**, and MF **(C)**. **(D)** KEGG enrichment analysis shows the enriched pathways of DEGs. The size of each point represents the gene counts, and the color represents the p-value. GO, Gene Ontology; KEGG, Kyoto Encyclopedia of Genes and Genomes; BP, biological process; CC, cellular component; MF, molecular function.

Four microarray datasets (GSE6344, GSE15641, GSE76351, and GSE781) were obtained from the GEO database, which is a repository for high-throughput gene expression data. Notably, GSE15641 contained 92 samples: 23 Normal, 32 clear cell RCC (ccRCC), 11 papillary RCC (pRCC), 6 chromophobe RCC (chrRCC), 12 Oncocytoma (OC), and 8 transitional cell carcinoma (TCC). In our study, 23 normal and 32 clear cell RCC (ccRCC) samples from GSE15641 were included for further analysis. Volcano plots were generated to show the gene expression profile data of the above four GEO datasets (**Figures 1A–D**). Overlapping differentially expressed genes (DEGs) that met the defined criteria (|log2 Fold Change|>1, P value<0.05) were screened and visualized by Venn diagram intersection analysis, including 240 upregulated genes and 132 downregulated genes (**Figures 1E, F**). Notably, the top 100 DEGs expression profiles of the four GEO datasets and their chromosomal locations are displayed in **Figures 1G, H**. To get deeper insights into the biological function of these DEGs, an online analysis website, DAVID was utilized to conduct Gene Ontology (GO) and Kyoto Encyclopedia of Genes and Genomes (KEGG) enrichment analyses with the threshold of a P value <0.05. The “GOChord” package was employed to visualize GO terms, which were divided into three categories: biological processes (BP), cellular component (CC) and molecular function (MF) (**Figures 2A–C**). The results for BP terms revealed that inflammatory response, response to hypoxia, angiogenesis and immune response were enriched across the overlapping DEGs. For CC terms, the remarkably enriched GO terms were Golgi membrane, endoplasmic reticulum, focal adhesion, whereas for MF were receptor binding, enzyme binding and heparin binding. As shown in **Figure 2D**, KEGG enrichment analysis suggested that phagosomes, the PI3K-Akt signaling pathway, the Rap1 signaling pathway and cell adhesion molecules (CAMs) were enriched.

### PPI Network Construction and Hub Genes Identification

**Figure 3:**
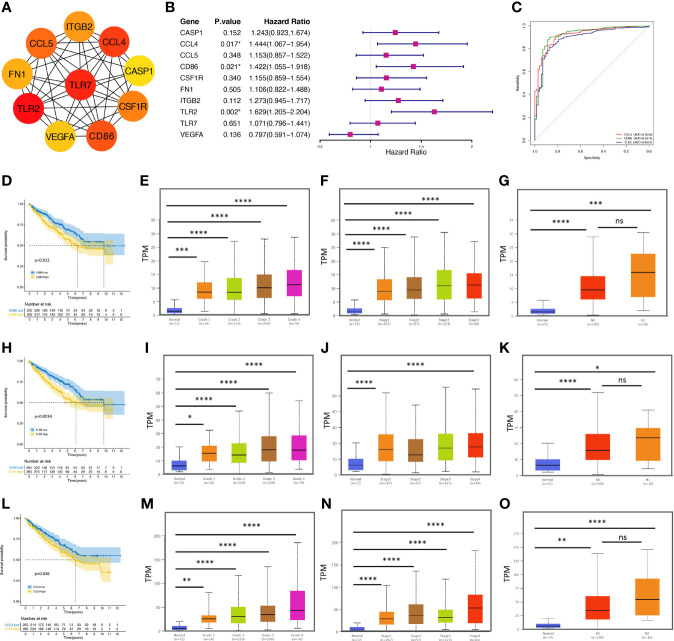
CCL4 serves as an immune-related prognostic biomarker and is correlated with clinicopathological characteristics. **(A)** PPI network of the top 10 hub genes evaluated by MCODE and cytoHubba of Cytoscape. **(B)** The forest plot shows the top 10 hub genes and their hazard ratios based on univariate Cox regression analysis. **(C)** ROC curve analysis shows the sensitivity and specificity of CCL4, CD86, or TRL2 in ccRCC diagnosis. **(D, H, L)** Kaplan–Meier analysis of CD86 **(D)**, TLR2 **(H)**, and CCL4 **(L)**. **(E–G)** Stratified analysis shows the correlations between CD86 and grade **(E)**, stage **(F)**, or metastasis **(G)** in ccRCC. **(I–K)** Stratified analysis shows the correlations between TLR2 and grade **(I)**, stage **(J)**, or metastasis **(K)** in ccRCC. **(M–O)** Stratified analysis shows the correlations between CCL4 and grade **(M)**, stage **(N)**, or metastasis **(O)** in ccRCC. PPI, protein-protein interaction; ROC, receiver operating characteristic. *p < 0.05; **p < 0.01; ***p < 0.001; ****p < 0.0001. ns, no significance.

To further explore the protein-protein interaction (PPI) networks among the overlapping DEGs, we imported the 240 upregulated and 132 downregulated genes into the STRING database and observed that 2133 edges and 366 nodes were involved in the PPI network analysis. From the perspective of a statistically significant combined score, we sent the network to Cytoscape for further exploration (**Supplementary Figure 1**). Molecule analysis among the hub genes were conducted with Cytoscape plug-ins (MCODE and cytoHubba). As shown in **Figure 3A**, the top 10 hub genes filtered out were Toll like receptor 2 (TRL2), Toll like receptor 7 (TLR7), C-C motif chemokine ligand 4 (CCL4), cluster of differentiation 86 (CD86), C-C motif chemokine ligand 5 (CCL5), colony stimulating factor 1 receptor (CSF1R), integrin subunit beta 2 (ITGB2), fibronectin 1 (FN1), vascular endothelial growth factor A (VEGFA) and caspase 1 (CASP1), which were screened by the MCC (Maximal Clique Centrality) algorithm. The differential expression of the top 10 hub genes in ccRCC samples compared with adjacent normal controls is shown in **Supplementary Figure 2**.

### Immune-Related Prognostic Biomarker Associated With Clinicopathological Characteristics in ccRCC

After we evaluated the proportional-hazards assumption (**Supplementary Figure 3**), univariate Cox regression analysis was performed to clarify the correlation between the overall survival (OS) and the hub genes (**Figure 3B**). The results suggested that three genes (CCL4, CD86, TRL2) were extracted negatively associated with OS, confirmed by the Kaplan-Meier survival method (**Figures 3D, H, L**), suggesting that these genes might serve as potential biomarkers associated with poor clinical performance in further analysis. To understand the accuracy and predictability of these three genes in prognosis (32), time-dependent receiver operating characteristic (ROC) curve analysis was used to employ the significance of CCL4, CDD86, and TRL2 in ccRCC prognosis. However, we observed no specific results. Interestingly, the results of a ROC curve analysis based on diagnosis showed that the area under the curve (AUC=0.9226) of CCL4 occupied the top position (**Figure 3C**), suggesting that CCL4 might have a great specificity and sensitivity in ccRCC diagnosis. Subsequently, we evaluated CD86 (**Figures 3E–G**), TLR2 (**Figures 3I–K**), and CCL4 (**Figures 3M–O**) transcriptional levels in the TCGA database. We observed that these three genes were positively correlated with stage, grade, and metastasis in ccRCC patients. However, some previous studies have uncovered the potential of CD86 and TLR2 in ccRCC progression, while the importance of CCL4 remains unknown. In addition, based on the intersection work of 457 immune-related genes downloaded from the ImmPort database, CCL4 was screened out to be the only gene as a potential immune-related prognostic biomarker in ccRCC.

### Comparison of KEGG Pathways Correlated With CCL4

**Figure 4:**
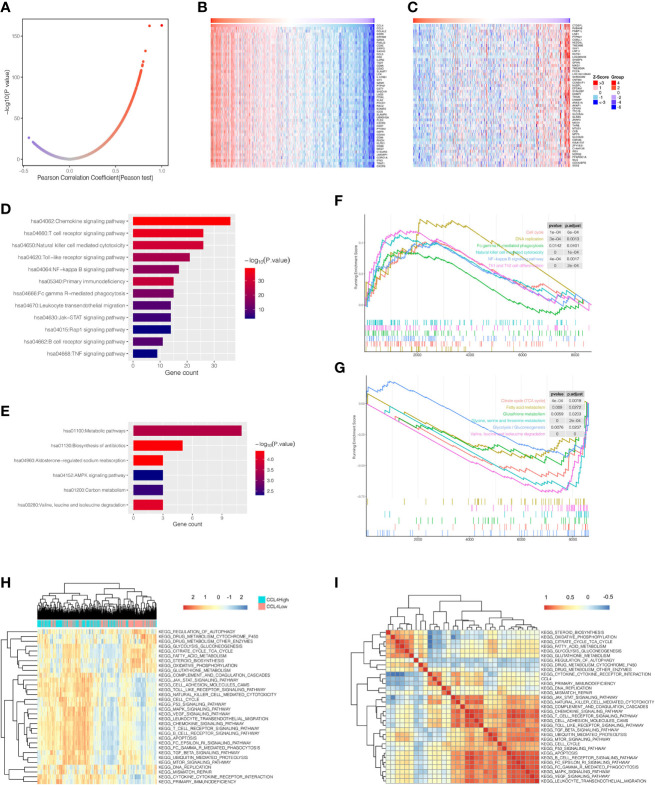
KEGG pathway analysis of CCL4. **(A)** Pearson correlation coefficient patterns of genes coexpressed with CCL4. **(B, C)** Heatmaps of gene sets positively **(B)**, and negatively **(C)**, correlated with CCL4. **(D, E)** KEGG pathways enriched in gene sets positively **(D)**, and negatively **(E)**, correlated with CCL4. **(F, G)** GSEA analysis shows enriched pathways of the high **(F)**, and low CCL4 expression groups **(G)**, using the median value of CCL4 as the cutoff. **(H)** Heatmap of KEGG pathways calculated in each sample based on ssGSEA algorithm. The color scale from blue to red indicates downregulation to upregulation. **(I)** Correlation matrix between CCL4 and enriched KEGG pathways. GSEA, Gene Set Enrichment Analysis; ssGSEA, single-sample Gene Set Enrichment Analysis.

Genes coexpressed with CCL4 are displayed as shown in a volcano plot (**Figure 4A**), as well as the heatmap (**Figures 4B, C**) using an online tool LinkedOmics (33). The genes were divided into two groups: gene sets positively or negatively correlated with CCL4. KEGG pathway analysis was conducted for the top 50 significant genes of each group, respectively (**Figures 4D, E**), and indicated that tumor proliferation and immune-related pathways were enriched in the positively correlated group, such as the NF-kB signaling pathway, the Jak-STAT signaling pathway, the natural killer cell mediated cytotoxicity, and the T cell receptor signaling pathway. Interestingly, the negatively group contained mainly metabolic activities like Valine, leucine and isoleucine degradation. To obtain deeper insights into biological functions associated with CCL4, we also employed a gene set enrichment analysis (GSEA) to uncover the potent pathways CCL4 enriched based on another stratified method, which divided all TCGA-KIRC samples into CCL4 high and low expression groups using the median value of CCL4 expression as the cutoff. As expected, the results tended to be mutually consistent as previously revealed (**Figures 4F, G**). Moreover, the gene set variant analysis (GSVA) based on single-sample gene set enrichment analysis (ssGSEA) algorithm was performed to quantify the enrichment levels of various biological processes in different samples based on stratification by CCL4 expression, which produced similar results with this different approach(**Figure 4H**), indicating the stability and repeatability of the findings. The correlation profiles between CCL4 and the ssGSEA score of KEGG pathways are shown in **Figure 4I**. In summary, KEGG enrichment analysis from different perspectives further confirmed that CCL4 might play crucial roles in immunomodulation and carcinogenetic processes in ccRCC patients.

### CCL4 Was Involved in TME Modulation in ccRCC

**Figure 5:**
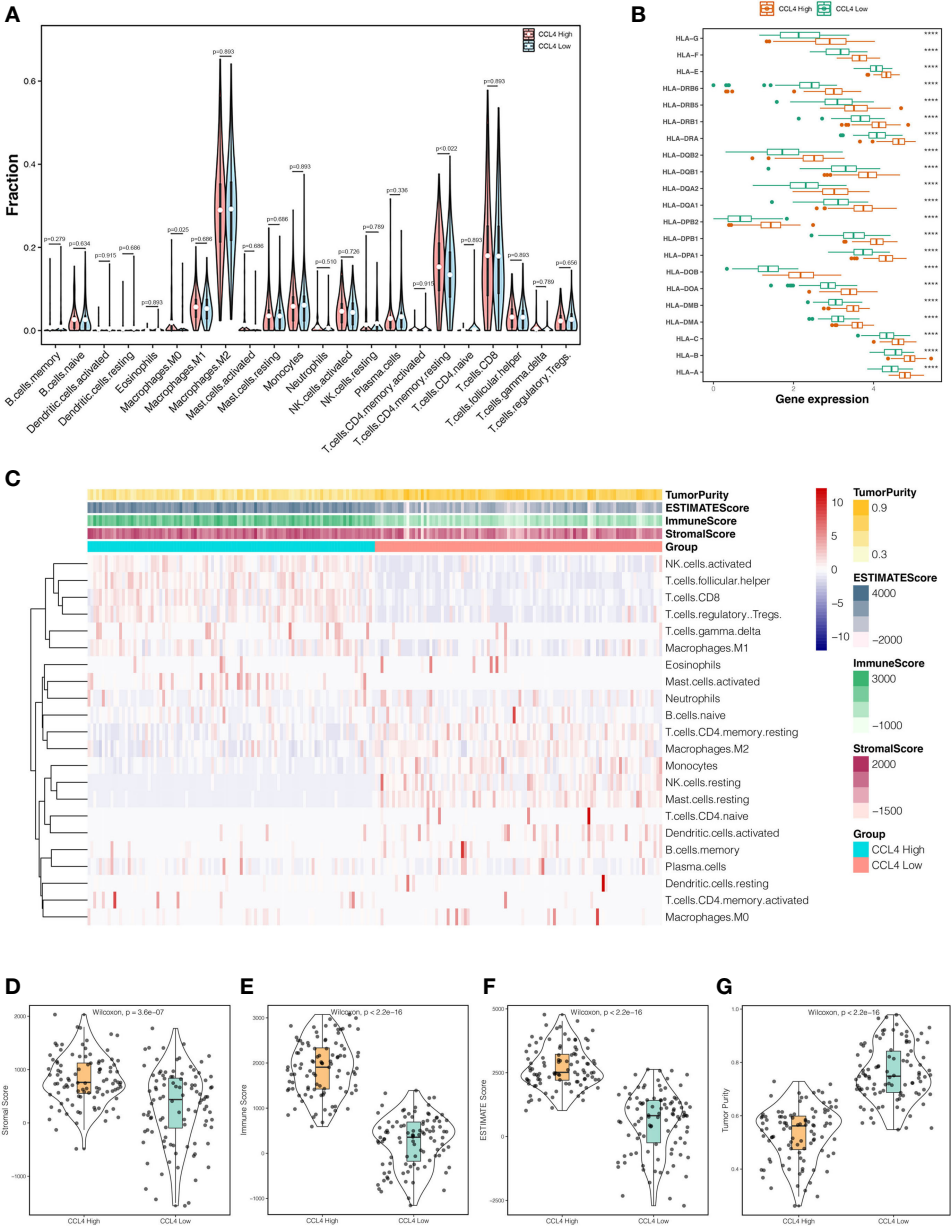
Interrelation between CCL4 and TME. **(A)** Violin plots represent the relative proportions of infiltrating immune cells in the CCL4 high and low groups using the CIBERSORT algorithm. **(B)** Comparison of HLA family genes expression between the CCL4 high and low groups. **(C)** Landscape of immune cell infiltration and TME characteristics in ccRCC samples. **(D–G)** Distributions of the stromal score **(D)**, immune score **(E)**, estimate score **(F)**, and tumor purity **(G)**, in the CCL4 high and low groups. TME, tumor microenvironment; ****p < 0.0001.

In the last decade, the TME has long flown under the radar in regard to its extraordinary status in clinical outcomes and therapeutic efficacy (15, 17, 18). To investigate whether CCL4 is involved in modulating the TME, we extended the “CIBERSORT” algorithm to estimate the relative infiltration proportion of 22 immune cell types from TCGA-KIRC samples, which were divided into CCL4 high and low groups according to the median value of CCL4 expression. As shown in **Figure 5A**, only M0 macrophage exhibited significantly higher immune infiltration levels in the CCL4 high groups. In addition, we also observed that HLA family genes were highly expressed in the CCL4 high groups (**Figure 5B**). In view of the above results, we concluded that high CCL4 expression might give rise to a high immune infiltration level. Subsequently, the TME characteristics of each sample in the CCL4 high and low groups deduced by the “estimate” package (**Figures 5C–F**) were analyzed. The results suggested that the TME scores of the two groups were significantly different and the CCL4 high group achieved higher estimate score, immune score and stromal score, while the CCL4 low group tended to be the opposite. In this study, the tumor purity of the CCL4 high group was lower than that of the low group (**Figure 5G**). Generally, this study uncovered that CCL4 was positively correlated with immune infiltration and TME characteristics.

### CCL4 Could Predict Immune Checkpoint Inhibitors Efficacy

**Figure 6:**
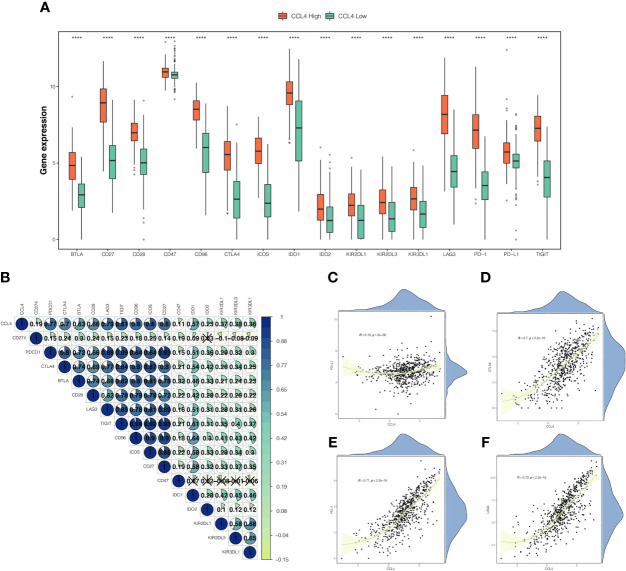
Correlation between CCL4 and immune checkpoints. **(A)** Comparison of immune checkpoint genes expression between the CCL4 high and low groups. **(B)** Correlation heatmap of CCL4 and immune checkpoint genes. The area and gradual color of the pie, as well as the number upon it correspond to the Pearson correlation coefficient. The cross indicates no statistically significance. **(C–F)** Scatter plots combined with density maps further show the correlations between CCL4 and PD-L1 **(C)**, CTLA4 **(D)**, PD-1 **(E)**, or LAG3 **(F)**. ****p < 0.0001.

To investigate the correlation between CCL4 and ICPs, we compared ICP expression between the CCL4 high and low groups and observed that ICPs were significantly and highly expressed in the CCL4 high group, particularly PD-L1 (**Figure 6A**), which was considered to be a predictive biomarker in anti-PD-1 directed therapy. Moreover, a correlation heatmap revealed that CCL4 was strongly correlated with common ICPs, including PD-1, CTLA4, LAG3 and TIGIT (T cell immunoreceptor with Ig and ITIM domains) (**Figure 6B**), indicating that CCL4 might be associated with ICI efficacy attributing to positive correlation with ICP expression. We also portrayed scatter plots combined density maps to further visualize the strong correlation between CCL4 and the commonly studied ICPs: PD-L1, CTLA4, PD-1 and LAG3 (**Figures 6C–F**). In view of the above results, it is reasonable to conclude that CCL4 may serve as an indicator for clinical use of immune checkpoint inhibitors (ICIs) and be conducive to predict ICI efficacy in ccRCC patients subjected to immunotherapy. Despite this, more experimental analyses are needed to validate the importance of CCL4 in ICIs stratification.

### Somatic Mutation Landscape and Tumor Mutational Burden Stratified By CCL4 Expression

**Figure 7:**
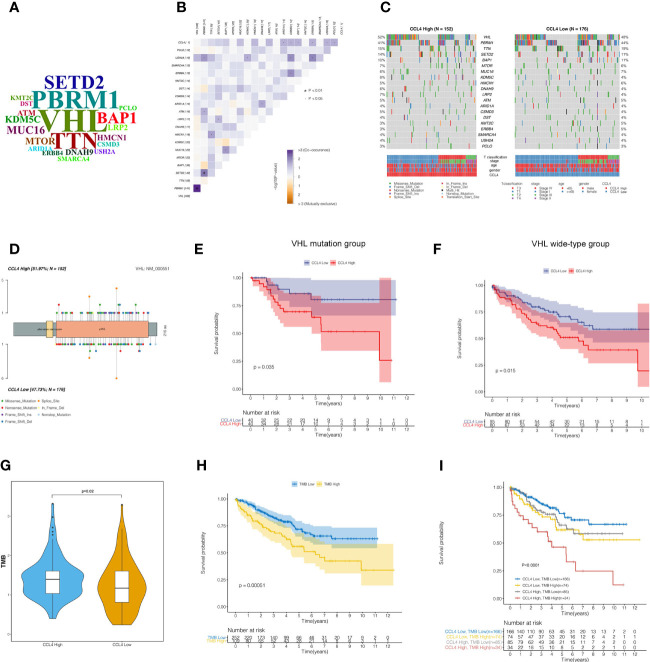
Somatic mutation landscape and tumor mutational burden in ccRCC samples. **(A)** WordCloud presentation of the top 20 mutated genes in ccRCC samples. The size of each gene indicates the relative mutational frequency. **(B)** Correlation heatmap shows cooccurring and mutually exclusive genes with CCL4 among the top 20 mutated genes. **(C)** Waterfall plots show the differential distribution profiles of the top 20 mutated genes in the CCL4 high and low groups, respectively. **(D)** VHL mutation sites in the peptide sequence of the CCL4 high and low groups are visualized in a lollipop diagram. **(E, F)** Kaplan-Meier analysis of the VHL mutant and wild-type groups shows obvious significance of the two groups stratified by CCL4 expression. **(G)** TMB score of the CCL4 high and low groups. **(H)** Kaplan-Meier analysis of the TMB high and low groups. **(I)** Kaplan-Meier analysis of four groups stratified by CCL4 expression and the TMB score. TMB, Tumor Mutational Burden.

Accumulating evidence has shown that tumor-specific mutational events can trigger the presentation of neoantigens, which are hopefully novel therapeutic targets (34, 35). To determine the impact of CCL4 expression levels on the somatic mutation landscape in 336 KIRC samples, the R package “maftools” was applied to find that 83.33% of the samples were detected to have at least one type of mutational events among the top 20 mutated genes in ccRCC (**Supplementary Figure 4**). WordCloud plots were created to represent the frequencies of the top 20 mutated genes (**Figure 7A**). Specially, as the previous studies reported, TTN out of the top 20 genes is rarely considered a tumor-related gene. Although TTN always ranks the top mutation gene lists, most missense mutations on TTN do not directly confer a selective growth advantage because it encodes more than 30000 amino acids and poses many mutations compared to other genes. Notably, this study generated fresh insight into cooccurring and mutually exclusive genes with CCL4 among the top 20 genes, indicating that ARID1A, CSMD3, DST, ERBB4, USH2A and PCLO mutations were significantly cooccurred along with CCL4 (**Figure 7B**). Subsequently, we divided 336 samples into CCL4 high and low expression groups based on stratification by CCL4 expression. The distinctive mutation distribution profiles of the top 20 mutated genes in the two groups are shown in **Figure 7C**. As detected by Fishier’s exact test with the threshold of P<0.05, there were no significant differences in the top 20 mutated genes based on this classification. Among the top 20 mutation events, VHL mutation occupied the top position, accounting for 52% and 48% of the frequent mutation distribution profiles within the two groups, respectively. Therefore, we portrayed the VHL mutation sites on the peptide sequence to visualize the translational effects of genetic mutations within the two groups by a lollipop diagram (**Figure 7D**). According to a previous study showing that the mutation of VHL gene plays critical roles in ccRCC pathogenesis and clinical outcome prediction (36), we conducted survival analysis based on the different VHL gene mutation status stratified by CCL4 expression, which suggested that high CCL4 expression corresponded to a high mortality rate in both the VHL mutant and wild-type cohorts (**Figures 7E, F**). Besides, our data illustrated that the CCL4 high group conferred to higher TMB levels (**Figure 7G**). According to a previous study, high somatic TMB levels correspond to the improved survival time in non-small-cell lung cancer (37). However, it still remains controversial whether TMB levels are associated with clinical outcomes in ccRCC. Therefore, we divided TCGA-KIRC samples into two groups according to the median value of the TMB score. Subsequent survival analysis told the significant differences between these two groups that patients with a low TMB score might have a superior survival probability compared to those with a high TMB score in ccRCC (**Figure 7H**). To further investigate the latent crosstalk between CCL4 and the TMB, we originally conducted survival analysis to examine whether CCL4 showed a distinct effect on clinical outcome in the background of similar TMB scores. As shown in **Figure 7I**, patients with low CCL4 expression and a low TMB score had prolonged survival time compared to those with high CCL4 expression and a low TMB score, and patients with high CCL4 expression and a high TMB score were associated with a poorer prognosis than those with low CCL4 and a high TMB score. When patients with high CCL4 expresssion were stratified by the TMB score, we also observed that the TMB score had a noticeable effect on the survival time of the patients with high CCL4 expression and similar results reappeared in the CCL4 low groups. Moreover, patients with low CCL4 expression and a low TMB score tended to encounter the best clinical outcomes than the other groups. Although we did not observe a significant distinction in tumor-specific mutations between the CCL4 high and low groups, we proposed that CCL4 could reflect the level of the TMB, on the other hand, the TMB might synergistically serve with CCL4 as a predominant prognostic signature related to novel therapeutic targets in ccRCC.

## Discussion

Although considerable evidence has highlighted the importance of the TME in cancer progression and immunotherapy efficacy, many studies have failed to distinctly clarify the molecular characteristics and enrichment pathways driven to modulating the TME. In this study, we extracted a potential immune-related biomarker CCL4 from high-throughput sequencing profiles that might promote aggressive tumor proliferation and be positively associated with immune infiltration levels and the immunotherapy response in ccRCC.

The integrated bioinformatic analysis revealed that high CCL4 expression was significantly associated with reduced survival time and advanced grade, stage, and metastasis within ccRCC, indicating that CCL4 may act as an oncogene in ccRCC. Subsequently, we performed functional enrichment analysis based on different statistical analyses. KEGG analysis showed that genes positively correlated with CCL4 were enriched in immune-related and carcinogenic pathways, while the opposite were related to metabolic activities. To verify this, we also conducted GSEA and GSVA analysis. Interestingly, we observed that both of results tied very well with previous findings, indicating that CCL4 might play a dual role in remodeling the TME through the transition from metabolism to immunity and carcinogenesis. According to a previous study, high CCL4 expression might stimulate the infiltration of tumor-specific macrophages in colon cancer (38). However, the correlation between CCL4 and immune cell infiltration in ccRCC remained unclear. Therefore, we evaluated the proportions of 22 immune cells in TCGA-KIRC samples using the CIBERSORT algorithm and calculated the TME score using the ESTIMATE algorithm. The results indicated that high CCL4 expression might give rise to high immune infiltration levels and be positively correlated with TME characteristics, excluding tumor purity. Previous studies uncovered that low tumor purity might lead to reduced survival time and an immunosuppressive TME, resulting in unfavorable clinical outcomes and low therapeutic efficacy (39–41). In this study, we concluded that low tumor purity in the CCL4 high groups might induce immunosuppressive TME, which contributes to antitumor immunity inhibition, activated immune checkpoint accumulation and a poor prognosis. In addition, we also observed that HLA family genes were highly expressed in the CCL4 high groups, including HLA-G that was reported to be a novel immune checkpoint molecule targeted by ICI treatment (42, 43), confirming that high CCL4 expression could not only induce high immune infiltration, but also imply a potential correlation with ICI therapeutic efficacy. To obtain deeper insight into the correlation between CCL4 and immunotherapy response, a correlation analysis was performed to demonstrate that CCL4 was positively related to some novel checkpoint genes, indicating that high CCL4 expression might serve as an indicator for ICI treatment response. Regardless, the question of whether CCL4 could guide ICIs for clinical application needs to be further extended through more clinical trials.

Previous studies have demonstrated that VHL loss-of-function mutations may induce angiogenesis and cell proliferation by evevating the expression of hypoxia-inducible factor (HIF) in ccRCC (44–47). Therefore, we displayed the somatic mutation landscape of the top 20 aberrantly mutated genes stratified by the expression of CCL4, in which VHL was the most frequently altered gene. However, Fishier’s exact test did not reveal significant distictions between different CCL4 expression groups based on the background of mutation events. A further astonishing finding was that in ccRCC patients, high CCL4 expression conferred unfavorable outcomes compared to low CCL4 expression in both the VHL-mutant and wild-type groups. Obviously, this hypothesis needs to be further confirmed by validation in a larger cohort. However, it still remains controversial whether tumor mutational burden (TMB) is positively correlated with immune infiltration or has noticeable impacts on the promotion of immunotherapy in various tumors (48–50). Zhang et al. reported that a high TMB score indicated low immune infiltration and poor clinical outcomes in ccRCC (48), while some studies (51, 52) demonstrated that high TMB levels could improve ICI efficacy (51, 53, 54). We compared TMB levels between the CCL4 high and low groups and observed that CCL4 high group conferred to a significantly higher TMB level. Subsequently, survival distribution based on stratified analysis of the TMB alone or synergistically with CCL4 revealed that prognostically relevant signatures comprising CCL4 and the TMB might shed new light on promising therapeutic targets in ccRCC. Contrary to the findings of Zhang et al, we concluded that high TMB levels conferred to higher immune infiltration, poorer prognosis, as well as promoted ICI efficacy owing to the positive correlation with CCL4 in ccRCC patients based on the above findings in our study.

## Conclusion

We concluded that CCL4 might promote tumor progression in ccRCC and serve as an immune-related prognostic biomarker to predict clinical outcomes and immunotherapy response. Furthermore, the present findings confirmed that CCL4 has profound impacts on tumor microenvironment remodeling, accounting for the status switch from metabolism to immunity and tumor proliferation, which may cast new lights on the crucial status of immune-related molecules involved in immunomodulation and tumor progression.

Nevertheless, further research is needed to investigate the underlying mechanism of the specific immune-related biological process CCL4 involved in remodeling tumor microenvironment characteristics and driving immunotherapy response. Moreover, it is necessary to confirm the present findings of integrated bioinformatics analysis through cytological experiments and clinical cohorts.

## Data Availability Statement

Publicly available datasets were analyzed in this study. This data can be found here: The Cancer Genome Atlas database (https://portal.gdc.cancer.gov/) and GEO (https://www.ncbi.nlm.nih.gov/geo).

## Author Contributions

Conceptualization, LZ and MZ. Methodology, LW, TY, and QS. Software, LZ. Validation, XL, MM, and NZ. Investigation, LZ, MJ, and RS. Writing—original draft preparation, LZ and MZ. Writing—review and editing, JL and JF. All authors contributed to the article and approved the submitted version.

## Conflict of Interest

The authors declare that the research was conducted in the absence of any commercial or financial relationships that could be construed as a potential conflict of interest.

## Publisher’s Note

All claims expressed in this article are solely those of the authors and do not necessarily represent those of their affiliated organizations, or those of the publisher, the editors and the reviewers. Any product that may be evaluated in this article, or claim that may be made by its manufacturer, is not guaranteed or endorsed by the publisher.
