# Supplementary material for: Identification of CCL4 as an Immune-Related Prognostic Biomarker Associated With Tumor Proliferation and the Tumor Microenvironment in Clear Cell Renal Cell Carcinoma
Source: Front Oncol. 2021 Nov 24;11:694664. doi: 10.3389/fonc.2021.694664 (PMC8652234; doi:10.3389/fonc.2021.694664)
Supplement: Supplementary file 1 [file DataSheet_1.docx]

Supplementary Material

**Supplementary Figures**


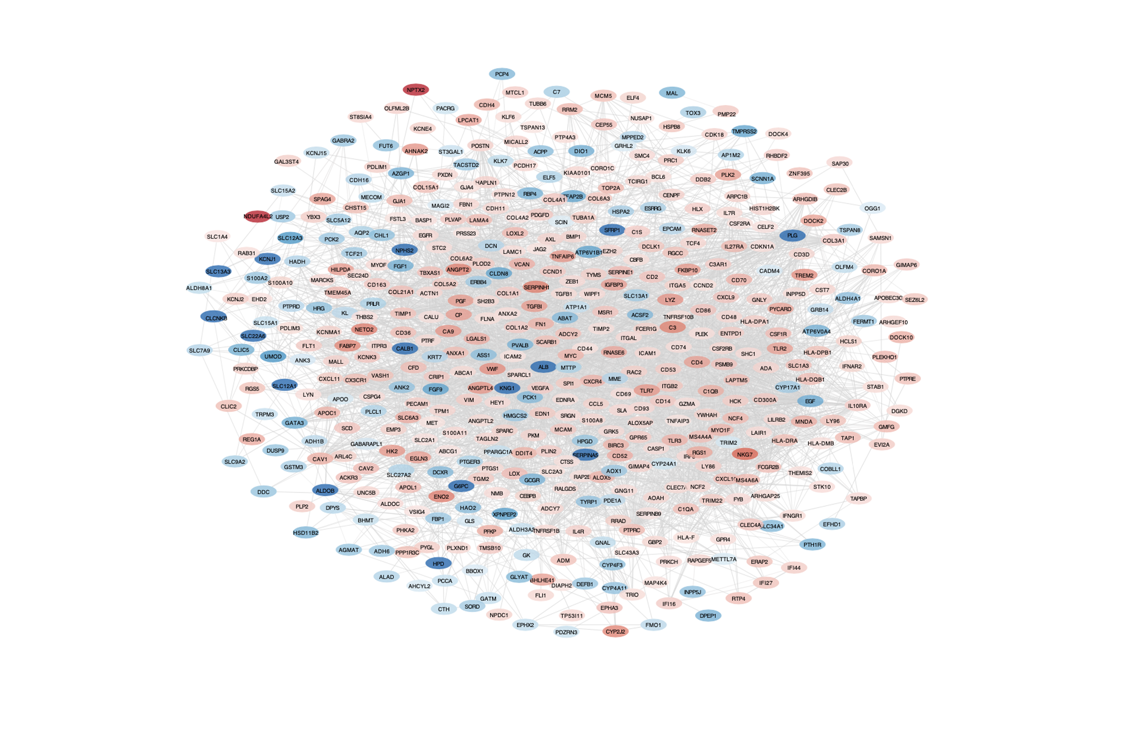


**Supplementary Figure 1 |** PPI network of DEGs created by Cytoscape. The color scale ranging from blue to red corresponds to value of log2FC, indicating downregulation to upregulation.

**
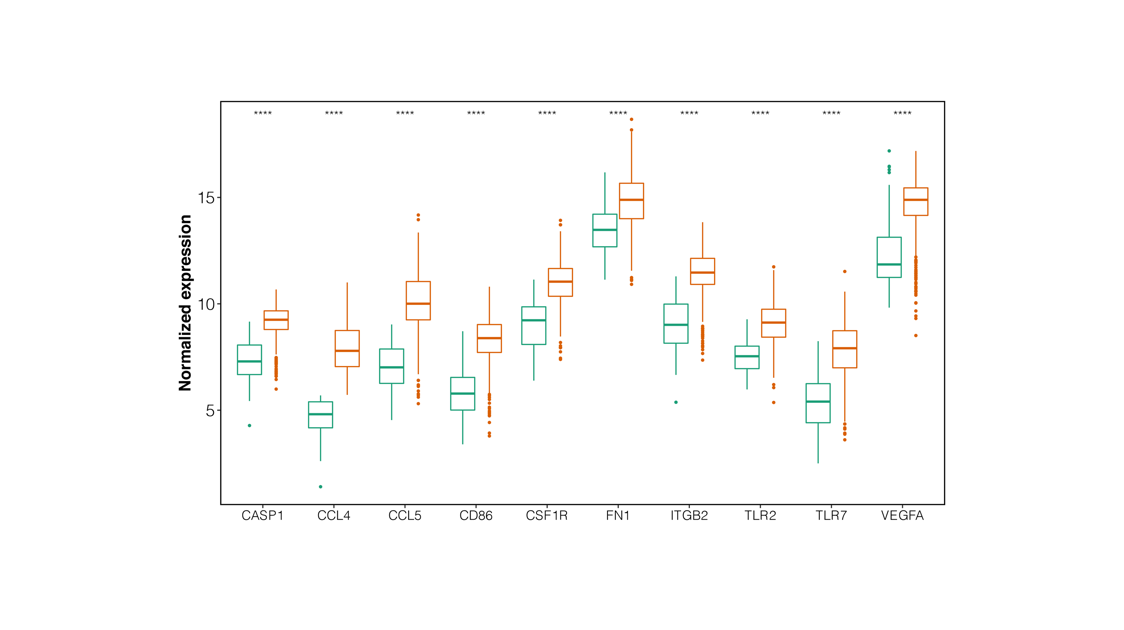
**

**Supplementary Figure 2 |** Comparison of the top 10 hub genes expression between tumor and normal controls in TCGA-KIRC samples.

**Supplementary Figure 3 |** Proportional hazard assumption of the top 10 hub genes in the Cox model.

**
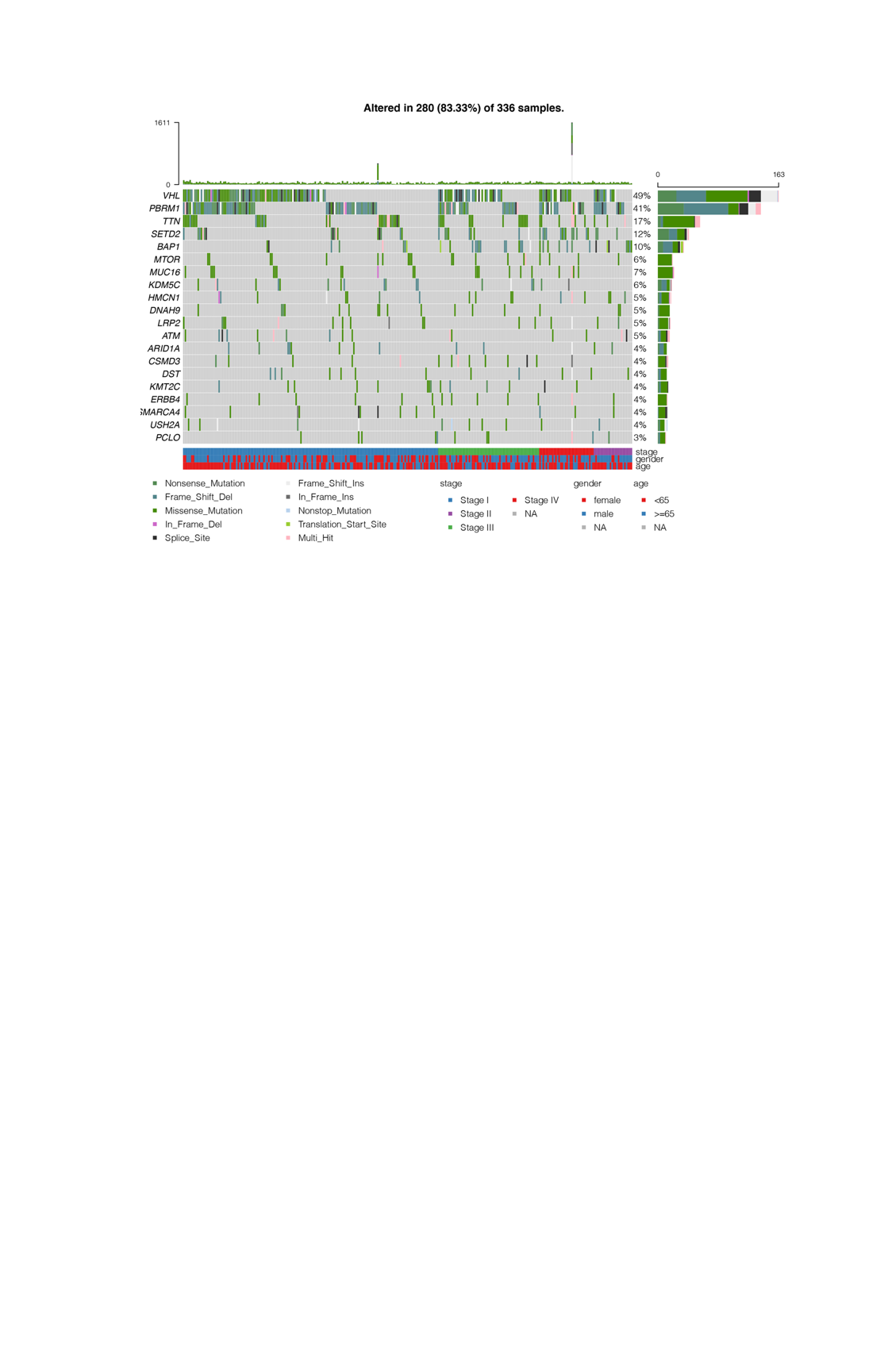
**

**Supplementary Figure 4 |** Somatic mutation distribution of top 20 mutated genes in ccRCC samples.
